# Background anaesthetic agents do not influence the impact of arginine vasopressin on haemodynamic states and cerebral oxygenation during shoulder surgery in the beach chair position: a prospective, single-blind study

**DOI:** 10.1186/s12871-017-0364-9

**Published:** 2017-05-30

**Authors:** Eun-A Jang, Ji-A Song, Ji Youn Shin, Jae Joon Yoon, Kyung Yeon Yoo, Seongtae Jeong

**Affiliations:** Department of Anesthesiology and Pain Medicine, Chonnam National University Medical School, 42 Jebong-ro, Dong-gu, Gwangju, 61469 South Korea

**Keywords:** Anaesthetic, Arginine vasopressin, Beach chair position, Cerebral oxygenation, Haemodynamics

## Abstract

**Background:**

Administration of arginine vasopressin (AVP) is associated with reducing jugular venous (SjvO_2_) and regional cerebral (rScO_2_) oxygen saturation under propofol-remifentanil (P/R) anaesthesia. We determined whether background anaesthetics modulate the effect of AVP on cerebral oxygenation and haemodynamics.

**Methods:**

We randomly allocated 60 adult patients scheduled for shoulder surgery in the beach chair position (BCP) into 4 groups, to receive either an intravenous bolus of saline (groups PR-S and SN-S) or 0.05 U/kg AVP (groups PR-AVP and SN-AVP) under P/R or sevoflurane-nitrous oxide (S/N) anaesthesia (*n* = 15 each). Haemodynamic variables, SjvO_2_ and rScO_2_ were measured.

**Results:**

AVP significantly increased mean arterial blood pressure (MAP) and decreased rScO_2_ in either anaesthetic group. AVP also decreased SjvO_2_ in the P/R groups but not in the S/N groups. The AVP-treated groups showed higher MAP and cerebral desaturation (>20% rScO_2_ decrease from baseline), along with lower HR and rScO_2_ in the BCP than those in the saline-treated groups. In contrast, AVP did not affect SjvO_2_ values or the incidence of SjvO_*2*_ < 50%. Baseline SjvO_2_ was lower and the magnitude of its reduction in the BCP was greater in the PR-AVP group than in the SN-AVP group, and the lowest SjvO_2_ values were 37 ± 6 and 57 ± 8%, respectively (*P* < 0.001).

**Conclusions:**

The choice of anaesthetic regimen did not affect cerebral oxygenation or haemodynamics of AVP in the BCP. However, the negative effect of AVP on cerebral oxygenation should be considered, especially under P/R anaesthesia.

**Trial registration:**

ClinicalTrials.gov identifier: NCT01687894, registered on September 18, 2012.

## Background

The beach chair position (BCP) induces hypotension and increases cerebral ischaemia risk during postural changes under general anaesthesia [1–5]. Ephedrine and phenylephrine are commonly used to maintain haemodynamic stability for anaesthesia-induced hypotension. However, their relatively short duration of action requires repeated administration or continuous infusion to raise the blood pressure when used in the BCP [6, 7]. Moreover, concerns have been raised regarding the effects of cerebral perfusion pressure (CPP) on cerebral oxygenation when α-agonists (phenylephrine or norepinephrine) are used [6, 8, 9], as they may reduce frontal lobe oxygenation.

Arginine vasopressin (AVP) has been used to correct hypotension in various clinical setting [10, 11]. AVP improved cerebral oxygenation and cerebral blood flow (CBF) in animal models [12, 13]. After changing to BCP, hypotension may persist for up to 30 min [1, 7], and AVP’s pressure effect lasts for about 30 min [11]. AVP may thus be an attractive agent for the treatment of hypotension associated with the BCP. However, despite the amelioration of BCP-induced hypotension, AVP given before positioning reduces jugular venous oxygen saturation (SjvO_2_) under propofol-remifentanil (P/R) anaesthesia [14, 15], suggesting a decrease in CBF possibly due to cerebral vasoconstriction. These studies also noted that AVP was associated with a reduction of the regional cerebral tissue oxygen saturation (rS_C_O_2_) [14, 15].

Background anaesthetics may influence the effect of vasopressors on cerebrovascular haemodynamics and autoregulation. Potent vasoconstrictors, such as phenylephrine and norepinephrine, increase CBF as estimated by transcranial Doppler under isoflurane, but not under propofol anaesthesia in healthy subjects [16]. Similarly, animals anaesthetised with isoflurane display significantly larger increases in CBF than those under total intravenous (IV) anaesthesia when exposed to phenylephrine and norepinephrine, although they show similar mean arterial pressure (MAP) and CPP responses [17]. Anaesthetic agents also have distinct effects on cerebral haemodynamics and metabolism [18, 19], which may have implications for the BCP. Volatile anaesthetics per se have a direct cerebral vasodilator effect and increase CBF relative to the cerebral oxygen demand [18]. Nitrous oxide also increases CBF but does not change cerebral metabolism in healthy volunteers [20], suggesting an arterial vasodilator effect.

In contrast, propofol maintains CBF well matched to the metabolic rate [19]. As such, oxygen delivery reserve appears greater in the BCP [9], and cerebral desaturation estimated by SjvO_2_ occurs less frequently during single lung ventilation [21] under sevoflurane- rather than propofol-based anaesthesia.

This study examined whether the choice of anaesthetic regimen influences the effect of AVP given as a prophylactic bolus on cerebral oxygenation and haemodynamics associated with the BCP. We hypothesised that the negative impact of AVP on cerebral oxygenation would be attenuated without affecting its haemodynamic effect during sevoflurane/nitrous oxide (S/N) anaesthesia. To determine cerebral oxygenation, we measured SjvO_2_ using an oximetry catheter and rS_C_O_2_ using near-infrared spectroscopy (NIRS).

## Methods

After receiving approval from the institutional ethics committee of our institution and informed consents, 60 patients (age, 19–70 years) scheduled for elective shoulder surgery in the BCP, were enrolled for this study. Patients who have American Society of Anaesthesiologists physical status ≥4, history of myocardial ischaemia, or pre-existing neurological diseases were excluded. The patients were randomly assigned to one of four study groups based on a computer-generated randomisation list: saline under P/R anaesthesia (PR-S, *n* = 15), AVP under P/R anaesthesia (PR-AVP, *n* = 15), saline under S/N anaesthesia (SN-S, *n* = 15) and AVP under S/N anaesthesia (SN-AVP, *n* = 15).

Patients were given 0.1 mg/kg of triazolam (Halcion^®^; Pfizer Korea, Seoul, Korea) for anxiolysis 1 h before arriving in the operating room. After arrival in the operating room, electrocardiography, noninvasive blood pressure and pulse oximetry were applied. After skin infiltration using 1% lidocaine, a 20-gauge radial arterial cannula was placed for continuous blood pressure monitoring and arterial blood gas analysis. The transducer was placed at the mid-axillary level when patients were supine and placed at the external auditory canal level when patients were in the BCP [22]. To assess the depth of anaesthesia, BIS^®^ A-2000™ (Aspect Medical Systems, Natick, MA, USA) was attached to the forehead, and an INVOS^®^5100B cerebral oximeter (Somanetics, Troy, MI, USA) was applied for monitoring of rS_C_O_2_. The two probes were placed at the forehead just above the bispectral index score (BIS) probe facing the medial margin of the other. After full pre-oxygenation, anaesthesia was induced, depending on the assigned group, with propofol (1.5–2 mg/kg) and remifentanil (1 μg/kg) (S/N group), or with 4 μg/mL of propofol and 3 ng/dL of remifentanil using a target-controlled infusion (TCI) pump (Orchestra Base Primea^®^; Fresinius, Brezins, France) (P/R group). After endotracheal intubation using IV administration of 0.8 mg/kg rocuronium, controlled ventilation was established at a tidal volume of 7 mL/kg and rate of 12/min with FiO_2_  =  0.5, and end-tidal carbon dioxide (ETCO_2_) was maintained between 35 and 40 mmHg by respiratory rate control. General anaesthesia was maintained with 50% N_2_O in oxygen at 3 L/min with sevoflurane in the S/N group, and with oxygen-enriched air (FiO_2_ 0.5) in the P/R group. Sevoflurane concentrations or effect-site propofol and remifentanil concentrations were adjusted to maintain BIS values at 40–50 throughout the surgery. A central venous catheter (PreSep™ Oximetry Catheter; Edwards Lifesciences, Irvine, CA, USA) was positioned under direct ultrasound visualisation, and the catheter tip was positioned to the jugular bulb contralateral side to operation site. SjvO_2_ was monitored using a Vigileo™ monitor (Edwards Lifesciences, Irvine, CA, USA).

After haemodynamics stabilised, IV bolus of 0.05 U/kg AVP (vasopressin; Han-Lim Pharmaceuticals, Seoul, South Korea) or an equal volume of saline was given over 20 s. The AVP dose was chosen based on our previous study that used AVP to prevent hypotension in the BCP [14]. The BCP was achieved (65–75° from horizontal) on a beach-chair table. The operation started about 20 min after the BCP had been realised and when haemodynamics became stable.

MAP, heart rate (HR), BIS and rS_C_O_2_ were recorded before induction of anaesthesia (room air). After induction of general anaesthesia, MAP, HR, BIS, rS_C_O_2_ and SjvO_2_ were recorded immediately before the administration of AVP or saline (baseline), before (time 0, during supine position after administration of AVP or saline) and after the patient was in the BCP. The recording interval was 1 min after the BCP had been reached for 15 min, and then every 5 min for additional 15 min. The maximum changes in SjvO_2_ were determined by calculating the differences between baseline and the lowest value of SjvO_2_ after BCP. SjvO_2_ values <50% for 5 min were regarded as jugular bulb O_2_ desaturation [23]. Cerebral O_2_ desaturation was defined as a > 20% reduction in rS_C_O_2_ from baseline for >15 s [24]. Hypotension was defined as MAP <50 mmHg and was treated with 8 mg of ephedrine injection with rapid fluid infusion. If hypotension persisted, additional dose of ephedrine (8 mg) was repeated every 2 min. At the end of surgery, residual neuromuscular block was antagonised with 15 mg of pyridostigmine and 0.4 mg of glycopyrrolate. Neurological and cognitive evaluations were also performed at a postoperative visit.

The primary outcome was the SjvO_2_ value after the postural change to the BCP with or without pretreatment with AVP. Secondary outcomes included changes in rS_C_O_2_ and haemodynamics (MAP and HR). In addition, the incidences of jugular bulb and cerebral oxygen desaturation and hypotension throughout the study (i.e. up to 30 min in the BCP) were also recorded.

### Statistical analysis

The primary endpoint was reduction of the SjvO_2_ in patients of the P/R group compared with those of the S/N group. To detect a difference in the lowest SjvO_2_ value of 10% observed within 15 min after the patient had been placed in the BCP between the groups, with an expected standard deviation (SD) of 10% and with a two-sided significance level α of 0.05 and a power of 0.9, a sample size of 13 individuals per group was required by “G power”. We recruited 15 patients in each group considering possible dropouts.

All values are expressed as mean ± SD or numbers. Data were analysed using StatView software ver. 4.0 (Abacus Concepts Inc., Berkeley, CA, USA). Patients’ demographic data and occurrences of side effects were analysed using one-way analysis of variance (ANOVA) or Pearson’s chi-square test. Continuous variables, including BIS, MAP, HR, rS_C_O_2_ and SjvO_2_, were analysed by a two-way repeated-measures ANOVA with time as a within-subject repeated measure and group (saline/AVP or PR-AVP/SN-AVP) as a between-subject variable. Post hoc pairwise test was performed using Dunnett’s *t*-test. A *p*-value <0.05 was considered significant.

## Results

**Fig. 1:**
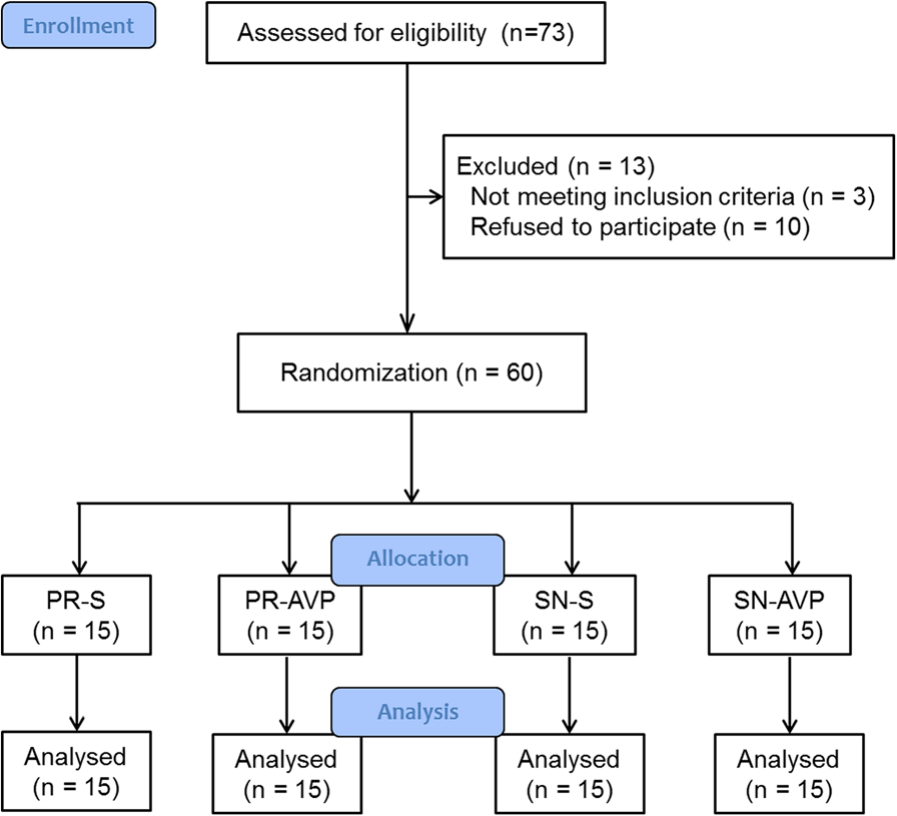
CONSORT flow chart showing the flow of patients through the trial.PR-S and PR-AVP groups are given saline and arginine vasopressin (AVP) under propofol-remifentanil (PR) anesthesia, respectively. SN-S and SN-AVP groups are given saline and AVP under sevoflurane-nitrous oxide (SN) anesthesia, respectively

Among the 73 patients assessed for eligibility, 60 provided written informed consent to participate. They were randomised into four groups and completed the study (Fig. 1). There were no significant differences in demographic data, sitting position time, blood loss, and fluid administration, between the four groups (Table 1).

**Table 1:** Demographic and intraoperative variables

	PR-S (*n* = 15)	PR-AVP (*n* = 15)	SN-S (*n* = 15)	SN-AVP (*n* = 15)	*P-*value
Gender (Male/female)	6/9	5/10	5/10	7/8	0.86
Age (yr)	62 (46–70)	59 (53–78)	62 (45–77)	65 (51–73)	0.45
Weight (kg)	64 ± 9	59 ± 10	62 ± 12	65 ± 11	0.45
Catheter placed (right/left)	6/9	3/12	4/11	6/9	0.56
Hemoglobin (g/dl)	13.7 ± 1.0	13.1 ± 1.3	13.7 ± 1.5	13.2 ± 1.3	0.51
Underlying diseases					
Hypertension	5	7	4	5	0.71
Diabetes	4	1	2	2	0.48
Preoperative medication					
β-blockers	1	2	2	1	0.84
Calcium channel blockers	2	4	3	5	0.62
ACEI or Ag II antagonist	2	2	2	3	0.95
Smoking history	3	2	2	1	0.77
Ephedrine administered					
Number of patients	11	4	6	3	0.01
Total dose per patient (mg)	15 ± 4	10 ± 4	13 ± 4	11 ± 5	0.14
Sitting position time (min)	151 ± 45	161 ± 29	181 ± 56	173 ± 34	0.24
Fluid administered (ml)	1690 ± 525	1773 ± 508	1600 ± 431	1920 ± 543	0.36
Blood loss (ml)	182 ± 154	142 ± 71	169 ± 116	116 ± 58	0.34

Data are mean ± SD or numbers.

PR-S: saline (S) under propofol-remifentanil (P/R) anesthesia; PR-AVP: arginine vasopressin (AVP) under P/R anesthesia; SN-S: S under sevoflurane-nitrous oxide (S/N) anesthesia; SN-AVP: AVP under S/N anesthesia; catheter: jugular venous catheter; ACEI: angiotensin converting enzyme inhibitor; AgII antagonist: angiotensin II antagonist

Table 2 presents the preoperative haemodynamics and intraoperative arterial blood gas data. None of these values differed significantly among the groups. No difference in BIS values was observed during surgery (44.5 ± 6.8 in PR-S, 44.8 ± 7.2 in SN-S, 46.3 ± 7.4 in PR-AVP and 45.8 ± 5.4 in SN-AVP group). The concentrations of intraoperative anaesthetic agents ranged 2.1–3.2 μg/ml of propofol and 2.0–3.7 ng/ml of remifentanil. End-tidal sevoflurane concentrations ranged between 1.1 and 2.0% during surgery. There were no neurologic complications in any patients during postoperative period.

**Table 2:** Preoperative hemodynamic and intraoperative arterial blood gas data

	PR-S (*n* = 15)	PR –AVP (*n* = 15)	SN-S (*n* = 15)	SN-AVP (*n* = 15)	*P-*value
Mean arterial pressure (mmHg)	104 ± 8	104 ± 10	101 ± 17	101 ± 16	0.88
Heart rate (beats/min)	67 ± 13	67 ± 12	67 ± 10	62 ± 13	0.46
SpO2 (%)	97 ± 2	96 ± 4	97 ± 2	97 ± 2	0.23
rSCO2 (%)	68 ± 4	66 ± 6	67 ± 6	66 ± 7	0.69
PaCO2 (mmHg)	38 ± 3	39 ± 4	39 ± 4	40 ± 3	0.56
PaO2 (mmHg)	199 ± 44	210 ± 41	210 ± 44	219 ± 49	0.71

Data are mean ± SD or numbers. PR-S: saline under propofol-remifentanil (P/R) anesthesia;PR-AVP: arginine vasopressin (AVP) under PR anesthesia; SN-S: saline under sevofluranenitrous oxide (S/N) anesthesia; SN-AVP: AVP under S/N anesthesia; SpO2: peripheral arterial saturation of oxygen; rSCO2: regional cerebral tissue oxygen saturation; PaCO2: arterial partial pressure of carbon dioxide; PaO2: arterial partial pressure of oxygen

**Fig. 2:**
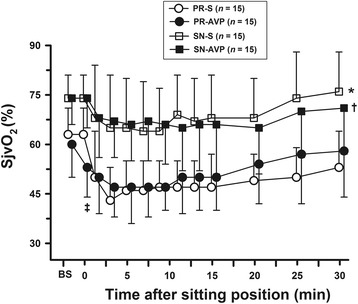
Effects of AVP on jugular venous oxygen saturation (SjvO_2_) after induction of anesthesia, before (presitting) and after the beach chair position. Data are means ± SD. BS represents the values after induction of anesthesia. Presitting values at time 0 were comparable between SN-S and SN-AVP groups, whereas they were significantly lower in PR-AVP than in PR-S groups (‡). *P < 0.05, compared with saline-given control groups; †*P* < 0.05, compared with AVP-treated groups by repeated measures two-way analysis of variance

Figure 2 shows the changes in SjvO_2_ values. SjvO_2_ (post-induction baseline) was comparable between the PR-S and PR-AVP groups and between the SN-S and SN-AVP groups before AVP was administered, but they were significantly lower in the PR groups than those in the SN groups (63 ± 8% in PR-S vs. 73 ± 7% in SN-S, *P* = 0.001; 61 ± 10% in PR-AVP vs. 73 ± 6% in SN-AVP, *P* = 0.0008). The SjvO_2_ values decreased significantly by 12% (relative change) after AVP in the PR-AVP group (*P* = 0.002), but remained unchanged in the SN-AVP group (*P* = 0.14), compared with respective post-induction baseline values. The SjvO_2_ values similarly decreased in the P/R and S/N groups while patients were in the BCP, regardless of AVP treatment. There was no significant effect of AVP in either the P/R- or the S/N-group (*P* = 0.11 and *P* = 0.31, respectively). However, the magnitude of the decreases of SjvO_2_ was greater in the P/R groups than those in the S/N groups (23 ± 7% in PR-S vs. 15 ± 6% in SN-S, *P* = 0.007; 25 ± 9% in PR-AVP vs. 15 ± 7% in SN-AVP, *P* = 0.003), with the lowest values within 15 min after adoption of the BCP (37 ± 6% in PR-AVP vs. 57 ± 8% in SN-AVP, *P* < 0.0001).

**Fig. 3:**
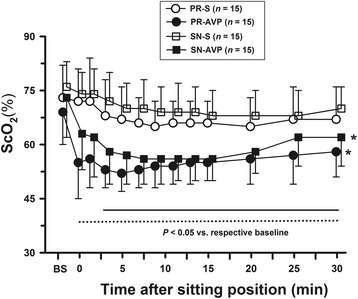
Effects of AVP on regional cerebral tissue oxygen saturation (rS_C_O_2_) after induction of anesthesia, before (presitting) and after the beach chair position. BS represents the values after induction of anesthesia. Presitting values in supine position are shown at time 0. Data are presented as mean ± SD. The solid and dotted lines indicate the time period during which rS_C_O_2_ differed from their baseline values in control and AVP groups, respectively (*P* < 0.05). *P < 0.05, compared with respective control groups by repeated measures two-way analysis of variance

Figure 3 shows the changes in the rS_C_O_2_ values. The rS_C_O_2_ values were similar after induction of anaesthesia (postinduction baseline) in the PR-AVP and SN-AVP groups. The rS_C_O_2_ values significantly decreased by BCP (all *P* < 0.05). The administration of AVP significantly decreased the rS_C_O_2_ in the P/R and S/N groups compared to their control (saline) groups (both *P* < 0.0001). However, there was no difference between P/R and S/N groups.

**Fig. 4:**
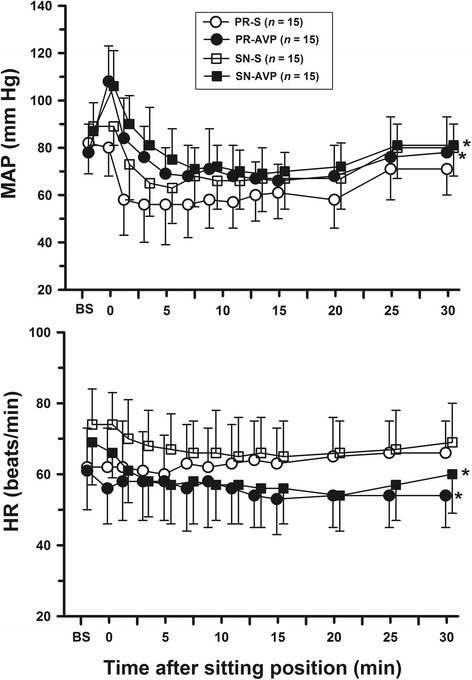
Effects of AVP on mean arterial pressure (MAP, upper) and heart rate (HR, lower) after induction of anesthesia, before (presitting) and after the beach chair position. BS represents the values after induction of anesthesia. Presitting values in supine position are shown at time 0. Data are presented as mean ± SD. *P < 0.05, compared with respective control groups by repeated measures two-way analysis of variance

Figure 4 shows the changes in the haemodynamic data. Anaesthesia induction decreased MAP in all groups (all *P* < 0.05). AVP then increased MAP significantly by 22–30% (*P* < 0.0001) without significant changes in HR in either anaesthetic group. Upon upright positioning, MAP decreased in all groups; however, the P/R and S/N groups showed a significant AVP effect on MAP (two-way ANOVA, *P* = 0.0003 and *P* = 0.04, respectively). In addition, the saline-treated groups developed early-onset hypotension after changing to the BCP and more episodes of hypotension requiring the administration of ephedrine compared with that in the AVP groups. The total ephedrine dose was higher in the PR-S group than that in the PR-AVP group (15 ± 4 mg vs. 10 ± 4 mg, *P* = 0.04), whereas it was not different between the SN-S and SN-AVP groups (*P* = 0.41). Anaesthetic induction did not alter HR in the P/R groups, but increased HR in the S/N groups (*P* < 0.05). HR decreased in all groups when patients were in the BCP, except for PR-S, in which it remained unaltered. Two-way ANOVA, however, revealed a significant AVP effect on HR in the P/R- and S/N-anaesthetic groups (*P* = 0.03 and *P* = 0.01, respectively).

Hypotension (MAP <50 mmHg) and the frequency of vasopressor therapy were lower in the PR-AVP than those in the PR-S group (*P =* 0.02). They also tended to be lower in SN-AVP than those in the SN-S group (*P =* 0.07). The incidence of cerebral desaturation was higher in the AVP-treated groups than in their respective saline-treated groups in both anaesthetic groups (each *P* < 0001). However, the incidence of SjvO_2_ < 50% and SjvO_2_ < 40% did not differ between the AVP- and saline-treated groups in either anaesthetic group. The incidence of SjvO_2_ < 50% in patients given saline (73% in PR-S vs. 13% in SN-S, *P* = 0.0009) was higher in the P/R group, as in patients given AVP (93% in PR-AVP vs. 13% in SN-AVP, *P <* 0.0001). Moreover, three patients (20%) in the PR-S and five patients (33%) in the PR-AVP group had SjvO_2_ < 40%, whereas none had SjvO_2_ < 40% in the SN-S and SN-AVP groups (*P* = 0.01).

## Discussion

The present study demonstrated that AVP pretreatment similarly reduced rS_C_O_2_ independent of the anaesthetic agent in patients undergoing shoulder surgery in the BCP. AVP per se before taking the BCP decreased SjvO_2_ in the P/R but not in the S/N group, consistent with previous studies [14, 15]. Moreover, the lowest SjvO_2_ values within 15 min in the BCP were significantly lower with a higher prevalence of jugular desaturation in the PR-AVP group than in the SN-AVP group. These results indicate that AVP for managing hypotension should be used cautiously in patients undergoing surgery in the BCP, particularly under P/R anaesthesia.

SjvO_2_ reflects the oxygen balance of the brain. The reduction in SjvO_2_ may reflect decreased CBF. Previous study showed that generalised cerebral vasoconstriction was developed after an injection of AVP into the cerebral circulation [25]. In another study, CBF autoregulation examined by using vasopressors was preserved under P/R anaesthesia, whereas it was adversely affected by isoflurane, allowing perfusion to become pressure-dependent in healthy patients [16]. The cerebral vessels probably constricted directly through a pharmacological effect and indirectly (through a reflex increase) by AVP given before the positioning; hence, CBF decreased to an extent that affected SjvO_2_ in the P/R but not in the S/N group. In fact, it has recently been demonstrated that AVP given before taking the BCP decreases SjvO_2_ in a dose-dependent fashion under P/R anaesthesia [15].

On the contrary, after the postural change to the BCP, AVP did not affect SjvO_2_ in either anaesthetic group, consistent with previous findings [14, 15]. Changes in cerebral oxygenation by AVP are controversial. It was reported that AVP decreased cerebral oxygenation, although AVP elevated systemic blood pressure under propofol anaesthesia [26]. In that study, the reduction of cerebral oxygenation in response to AVP did not redeem, suggesting that reduction of CBF was sustained. These findings are consistent with a prior demonstration of a reduced SjvO_2_ following AVP treatment before taking the BCP under P/R anaesthesia [14, 15].

In contrast, AVP yields favourable cerebral results in cases of unstable haemodynamic status, such as during cardiopulmonary resuscitation [12] or multiple trauma [13]. CBF and cerebral oxygenation may be increased by AVP when MAP was below the lower limit of cerebral autoregulation. It is likely that AVP shifted the blood to the brain by increasing systemic vascular resistance while in the BCP, resulting in comparable SjvO_2_ values between the saline- and AVP-treated groups. AVP may improve cerebral oxygenation when systemic haemodynamics and CPP are inadequate, such as during rapid surgical positioning into BCP.

Although AVP had no significant effect on SjvO_2_ in the BCP, its use was associated with significantly lower rS_C_O_2_ values and a higher incidence of cerebral desaturation in the BCP independent of anaesthetic agent. NIRS assumes a fixed cerebral arterial to venous volume ratio [27]; therefore, the composition of blood may affect cerebral oximetry. Sato et al. demonstrated that cerebral arterioles were constricted when CPP was increased by phenylephrine [6]. This indirect autoregulatory vasoconstrictive effect and direct cerebral vasoconstriction induced by AVP [25] may have induced a smaller arterial and larger venous contribution to the NIRS signal, resulting in a reduced rS_C_O_2_ [28].

It has also been shown that rS_C_O_2_ values measured by NIRS do not solely reflect oxygen saturation of the brain [8, 29]. AVP has more constrictor effects in the skin, skeletal muscle and splanchnic circulation, and less in the brain and heart [11, 13], so that blood flow should be diverted to the brain and heart at the same MAP. In this context, the discrepancy between SjvO_2_ and rS_C_O_2_ may be accounted for by a reduced cerebral arterial to venous volume ratio and/or reduced extracerebral blood flow [11], which may produce changes in rS_C_O_2_ without alterations in SjvO_2_. Similar observations have been made for other potent vasoconstrictors (phenylephrine or norepinephrine) [29]. In fact, rS_C_O_2_ is only weakly correlated with SjvO_2_, using SjvO_2_ as the standard reference, in the BCP [7]. Therefore, in contrast to SjvO_2_, rS_C_O_2_ measured using NIRS may not exactly reflect changes in CBF (perfusion) after the administration of vasopressors (i.e. AVP, α-agonists).

Renin-angiotensin system (RAS) inhibitors are increasingly used in patients scheduled for operation. The preoperative use of RAS inhibitors was associated with a higher occurrence of refractory hypotension in patients under general anaesthesia [30, 31]. In patients treated with RAS inhibitors, general anaesthesia blunts the sympathetic nervous system with inhibition of RAS [10]. Vasopressin has been proposed as the vasopressor of choice for treating refractory hypotension in those patients because it has different action mechanism [10]. In the present study, despite prophylactic use of AVP, two (40%) patients taking RAS inhibitors (1 of 2 in PR-AVP group, 1 of 3 in SN-AVP group) developed hypotension after BCP positioning. Nevertheless, patients given AVP had fewer hypotensive episodes and thus less chances of vasopressor therapy compared with control patients who received saline, justifying the use of AVP.

SjvO_2_ < 50% was an indication of cerebral hypoperfusion, and SjvO_2_ < 40% was associated with cerebral ischemia [23]. In the present study, SjvO_2_ values after induction of anaesthesia (baseline) were significantly lower in P/R groups than S/N groups, as reported previously [7, 19, 21]. In addition, the decrease in SjvO_2_after placing the patient in the BCP was more pronounced and thus jugular desaturation occurred more frequently in the PR-AVP group than in the SN-AVP group. Moreover, AVP per se given before positioning decreased SjvO_2_ in the P/R but not in the S/N group, as observed in previous studies [14, 15], suggesting that AVP decreased CBF to an extent that affected SjvO_2_ under P/R anaesthesia. Several other adverse effects were reported after the use of AVP: myocardial ischaemia, mesenteric ischaemia, digital ischaemia, and skin necrosis [11]. Although there was no patient showed cerebral complications with either anaesthesia, AVP should be used cautiously until its effects on the perfusion of vital organs including the brain are more defined, particularly under P/R anaesthesia.

The present study had several limitations. Because all patients in our study had no cerebral pathology, it is not certain how SjvO_2_ and rS_C_O_2_ will respond after changing to the BCP with AVP pretreatment in patients with impaired CBF autoregulation or cerebrovascular disease. In our study, most SjvO_2_ catheters were placed in the left side without an angiography examination, although the right jugular vein was the dominant drainage system in the majority of patients. The difference of the catheterisation sites may affect the results. Additionally, NIRS is affected by skin blood flow [8, 29]. We did not quantify skin blood flow, though AVP may decrease it [11]. Therefore, we could not differentiate changes in the NIRS signals between skin flow and CBF. Finally, AVP was administered 2 min before placing the patient in the BCP because its effect has been known to peak 2 min after injection [11]. Therefore, MAP in supine position was high, though it was still within the range of CBF autoregulation.

## Conclusions

Anaesthetic choice (S/N or P/R anaesthesia) did not influence the effect of AVP on cerebral oxygenation or haemodynamics in patients undergoing shoulder surgery in the BCP. SjvO_2_ was less preserved and jugular desaturation was more prevalent with P/R than with S/N anaesthesia, regardless of AVP use. Thus, the negative effect of AVP on cerebral oxygenation needs to be considered, particularly under P/R anaesthesia. Further studies in a larger population are required to establish the efficacy and safety of AVP for preventing hypotension while patients are in the BCP.

## Abbreviations

ACEIs: Angiotensin converting enzyme inhibitors
AIIRAs: Angiotensin II receptor antagonists
AVP: Arginine vasopressin
BCP: Beach chair position
CBF: Cerebral blood flow
CPP: Cerebral perfusion pressure
HR: heart rate
MAP: Mean arterial pressure
N_2_O: Nitrous oxide
NIRS: Near-infrared spectroscopy
RAS: Renin-angiotensin system
rScO_2_: Regional cerebral tissue oxygen saturation
SjvO_2_: Jugular venous oxygen saturation
